# The Extended Course of Study in Medical Colleges

**Published:** 1888-11

**Authors:** 


### *THE EXTENDED COURSE OF STUDY IN MEDICAL COLLEGES.*

The agitation of the important subject of advanced education in medicine has, of late years, engaged the serious attention of the intelligent members of the profession in this country. Our institutions, as well as our customs, are outgrowing the conditions of the past. New countries are compelled to improvise for the urgent necessities arising out of their rapid growth and development. The profession of medicine in this country has passed through many phases of its early developmental life. The instruction imparted in medical colleges in the first half of the present century was crude, when compared with that of the present day. Progress in medical science explains much of this improvement. Advance in wealth and culture has been, and is, a factor in the solution of this problem. The science of to-day cannot be grasped in the brief period allotted to the student of even a decade of years ago. The medical schools are compelled to yield to this demand. The first step is a lengthened session, and this advance is followed by the addition of another year, and of further studies to their curriculum.

The present year demonstrates that this necessity is recognized by our leading schools. The example established by a few of the medical colleges, notably the Medical Department of Niagara University in this city, the College of Physicians and Surgeons, now the Medical Department of Columbia College, in New York, and the University of Pennsylvania, will exert a strong influence in urging other schools to add to their curriculum and to their term of study.

The prospective establishment of the Department of Medicine in connection with Johns Hopkins University, with a course of five years, will constitute the most important epoch in the history of medical education in this country. A large endowment, which enables its trustees to command the best talent which this country affords, as in the selection of Professor Osler, of the University of Pennsylvania, for the Professorship of Clinical Medicine, which we learn has been accepted by that accomplished clinician, is a guarantee that it is their intention to found a school of medicine which will have no equal in this country and no superior in Europe.

Many medical colleges yet maintain the two-year standard, and send forth annually their graduates who have obtained the little knowledge of medicine they possess in this brief period. Cheap and limited instruction results in cheap and narrow doctors. The country is flooded with this material. The practice of medicine is degraded to a trade, with the commercial principle as the controlling motive. There is so much of this laxity in the profession that one would despair of a permanent reform if evidences of a higher sentiment did not crop out here and there.

This subject is one of vital importance. It needs a more general and widespread recognition in the profession and by the public. At present the medical degree carries with it no weight or influence beyond the individual merit and ability of its possessor. Our much-vaunted professional dignity and honor is a source of ridicule among the masses. What is needed is highly educated men, who labor in medicine for the love of scientific investigations. The schools can furnish them only by raising their standard, then lengthening their course and term of study, and thus make their diploma a certificate which will command respect at home as well as abroad.

